# Evacuation of the Pleural Cavity With an Infant Feeding Catheter Following en Bloc Resection of Hepatocellular Carcinoma and Involved Diaphragm–an Institutional Experience

**DOI:** 10.1155/1998/87539

**Published:** 1998-12

**Authors:** C. C. Chung, W. Y. Lau, C. K. Leow, K. L. Leung, P. B. S. Lai

**Affiliations:** Department of Surgery The Chinese University of Hong Kong Prince of Wales Hospital, Shatin New Territories Hong Kong

## Abstract

En bloc resection of hepatocellular carcinoma and the involved diaphragm will, towards the end of operation, require evacuation of the pleural cavity, usually with a chest drain. We describe our method and experience of evacuating the pleural cavity, at the time of diaphragmatic repair, with an infant feeding catheter without the need of a chest drain. We have found the method safe and efficacious.


*HPB Surgery*, 1998, Vol. 11, pp. 71-74
Reprints available directly from the publisher
Photocopying permitted by license only

© 1998 OPA (Overseas Publishers Association) N.V.
Published by license under
the Harwood Academic Publishers imprint,
part of The Gordon and Breach Publishing Group.
Printed in Malaysia.

### Evacuation of the Pleural Cavity with an Infant Feeding Catheter Following en Bloc Resection of Hepatocellular Carcinoma and Involved Diaphragm - an Institutional Experience

C. C. CHUNG[a], W. Y. LAU [a,*], C. K. LEOW[c], K. L. LEUNG[d] and P. B. S. LAI[a]

[a] *Medical Officer,* [b] *Professor of Surgery,* [c] *Associate of Professor,* [d] *Senior Medical Officer, Department of Surgery, The Chinese University of Hong Kong, Prince of Wales Hospital, Shatin, New Territories, Hong Kong*

*(Received 20 March 1997)*

**En bloc resection of hepatocellular carcinoma and the involved diaphragm will, towards the end of operation, require evacuation of the pleural cavity, usually with a chest drain. We describe our method and experience of evacuating the pleural cavity, at the time of diaphragmatic repair, with an infant feeding catheter without the need of a chest drain. We have found the method safe and efficacious.**

*Keywords:* Diaphragm, hepatocellular carcinoma, en bloc resection

### INTRODUCTION

Hepatocellular carcinoma (HCC) is a common malignancy worldwide [1, 2]. With better case selection, more refined surgical technique [3, 4] and optimal postoperative care [4, 5], the previously high mortality and morbidity of elective liver resection have given way to a more acceptably low level with satisfactory functional outcome. This, coupled with the grave prognosis of inoperable disease [2], makes hepatobiliary surgeons to adopt an aggressive attitude towards the choice of treatment options for hepatocellular carcinoma.

Local invasion of the diaphragm by HCC is not uncommon [6-8]. Even with diaphragmatic invasion there is still a good chance of cure if the lesion is amenable to en bloc resection [6, 7]. We present our experience of en bloc diaphragmatic resection for HCC and repair of the diaphragmatic defect without the need of inserting a chest tube.

### MATERIALS AND METHODS

Through a bilateral subcostal incision with a xiphoid extension, the falciform ligament is divided and a Gray's retractor (Stenning, Australia) is placed for optimal exposure.

*Corresponding author.

The right liver is mobilized by dividing the coronary and triangular ligaments. At the site where the diaphragm is invaded by the HCC, the diaphragm is circumferentially incised 1 cm from the edge of the tumour. A minimum of 1 cm rim of diaphragmatic tissue is left around the tumour and the tumour together with the attached diaphragmatic tissue is resected en bloc with the right liver in the usual manner. After ensuring that the hepatic transectional wound is dry, the diaphragm is repaired and closed with a single layer of continuous 1 nylon suture. One end of a 3.5 Fr infant feeding catheter (38 cm long) is placed inside the pleural cavity through the suture line and the other end of the catheter is placed under water seal within a gallipot (Fig. 1). After the diaphragmatic suture has been completed and just before the suture is tied, the anaesthetist is requested to manually hyperinflate the lungs. While the anaesthetist is maintaining full inflation, the suture is tied and simultaneously the infant feeding catheter is withdrawn by the assistant. A Robinson drain is placed in the space previously occupied by the right liver and the abdominal wound closed.

Postoperatively, the patient is nursed in the intensive care unit. Chest X-ray is taken routinely to assess the amount of residual pneumothorax.

We have employed this technique in resecting HCC with diaphragmatic involvement since 1989. The results were retrospectively reviewed.

### RESULTS

From November 1989 to January 1996, 22 patients with hepatocellular carcinoma and diaphragmatic invasion were operated. There were 19 males and 3 females, with a mean age of 49.5 (SD ± 14.3). Liver resections performed are shown in Table I. Twenty patients underwent resection with curative intent whereas palliative resection was performed for the remaining two patients. All patients had the diaphragm resected and repaired as described above.

None of these patients showed residual pneumothorax or pleural effusion on an immediate postoperative chest X-ray. Three patients (14%) developed a significant post-

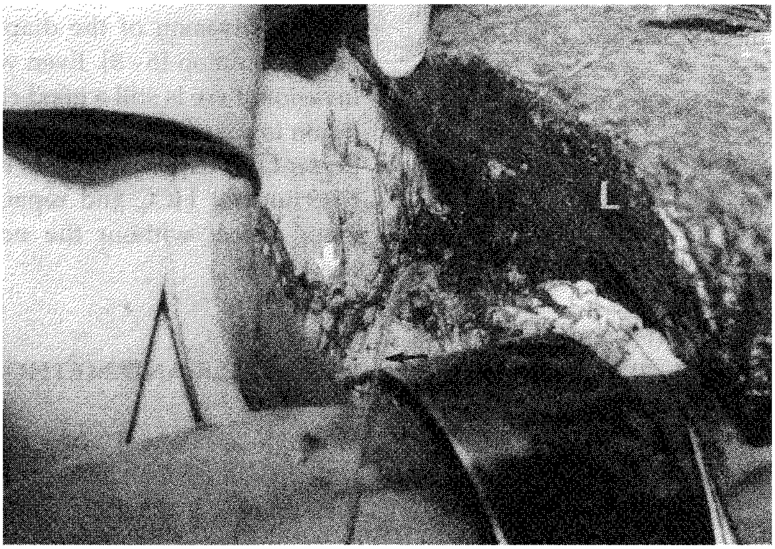

FIGURE 1 Following en bloc resection of the right hepatic lobe and the diaphragm (D), the diaphragmatic defect is closed with a single layer of continuous 1 nylon suture. One end of the infant feeding catheter (black arrow) is within the pleural cavity. [White arrow = suture line along the diaphragm; *L* = resected surface of the left lobe of a cirrhotic liver].

TABLE I Types of operation for the 22 patients

| Operation performed | Number (total = 22) |
|---|---|
| Right hepatectomy | 10 |
| Extended right hepatectomy | 4 |
| Resection of one or two segments | 4 |
| Non-anatomical resection | 3 |
| Right hepatectomy and right nephrectomy | 1 |

operative right pleural effusion requiring intervention. In 2 of these patients the effusion was evident on a chest X-ray taken on day 7, whereas in the third patient the effusion was detected on day 11. In all 3 patients the effusion settled rapidly after a single pleural tap. A continuous chest drain was not required.

There were 3 deaths. One patient died of multiorgan failure on day 2 after reexploration for postoperative bleeding from the hepatic wound. The other 2 patients died from progressive liver failure on day 10. The death of these 3 patients was not attributable to the diaphragmatic excision and mode of repair.

### DISCUSSION

Local invasion of the diaphragm by hepatocellular carcinoma is difficult to diagnose preoperatively. Preoperative ultrasonography, computed tomography and hepatic angiography are not helpful [6], and the only clue may be the presence of a hump distorting the right hemidiaphragm on a plain chest radiograph [6].

Surgery is difficult in this situation because the tumour usually involves the superior segments (segment VII and VIII) of the liver [9]. Moreover, the diaphragm is usually very vascular as a result of tumour collaterals and associated portal hypertension [7]. A transdiaphragmatic approach has been advocated [8], but it has never gained popularity because of the morbidity associated with a thoracotomy, notably the respiratory complications [10].

When the pleural cavity is entered through the diaphragm at operation, the conventional management is insertion of a chest tube and repair of the diaphragm. However, chest tube drainage is not without complications [11–13] and certainly a worsening pneumothorax can easily go unnoticed should a chest drain become blocked or kinked. In fact Stimpson *et al.*, showed that entry into the right pleural cavity followed by intercostal chest tube drainage incurred serious respiratory morbidity even without a formal thoracotomy [10].

Chest tube insertion is mandatory if a patient has had any form of lung parenchymal surgery or accidental lung injury at operations. If only the pleural cavity is breached at operation, chest tube insertion is not always necessary. We have found it safe and efficacious to evacuate the pleural cavity of our patients with intact lung parenchyma by the method described. This saves the patients from having a chest tube with its attendant discomfort and potential complications.

### *References*

[1] MacIntosh, E. L. and Minuk, G. Y. (1992). Hepatic resection in patients with cirrhosis and hepatocellular carcinoma. *Surg. Gynecol. Obstet.*, **174**, 245–254.

[2] Shiu, W., Dewar, G., Leung, N., Chan, M., Tao, M., Lui, C., Chan, C. L., Lau, W. Y., Metreweli, C. and Li, A. K. C. (1990). Hepatocellular carcinoma in Hong Kong: Clinical study on 340 cases. *Oncology*, **47**, 241–245.

[3] Lau, W. Y., Arnold, M., Guo, S. K. and Li, A. K. C. (1992). Microwave tissue coagulator in liver resection for cirrhotic patients. *Aust. N. Z. J. Surg.*, **62**, 576–581.

[4] Matsumata, T., Kanematsu, T., Shirabe, K., Sonoda, T., Furuta, T. and Sugimachi, K. (1990). Decreased morbidity and mortality rate in surgical patients with hepatocellular carcinoma. *Br. J. Surg.*, **77**, 677–680.

[5] Paquet, K. J., Kanssouris, P., Mercado, M. A., Kalk, J. F. R., Muting, D. and Rumbach, W. (1991). Limited hepatic resection for selected cirrhotic patients with hepatocellular and cholangiocarcinoma: a prospective study. *Br. J. Surg.*, **78**, 459–462.

[6] Lau, W. Y., Leung, K. L., Leung, T. W. T., Liew, C. T., Chan, M. and Li, A. K. C. (1995). Resection of hepatocellular carcinoma with diaphragmatic invasion. *Br. J. Surg*, **85**, 264–266.

[7] Jeng, K. S., Chen, B. F. and Lin, H. J. (1994). Enbloc resection for extensive hepatocellular carcinoma: is it advisable? *World J. Surg.*, **18**, 834–839.

[8] Shimada, M., Matsumata, T., Taketomi, A., Shirabe, K., Yamamoto, K., Itasaka, H. and Sugimachi, K. (1995). A new approach for liver surgery. Transdiaphragmatic hepatectomy for cirrhotic patients with hepatocellular carcinoma. *Arch. Surg.*, **130**, 157–160.
[9] Franco, D., Bonnet, P., Smadja, C. and Grange, D. (1985). Surgical resection of segment VIII (anterosuperior subsegment of the right lobe) in patients with liver cirrhosis and hepatocellular carcinoma. *Surgery*, **98**, 949–953.
[10] Stimpson, R. E., Pellegrini, C. A. and Way, L. W. (1987). Factors affecting the morbidity of elective liver resection. *Am. J. Surg.*, **153**, 189–196.
[11] Campbell, P., Neil, T. and Wake, P. N. (1989). Horner's syndrome caused by intercostal chest drain. *Thorax.*, **44**, 305–306.
[12] Beese, E. and Broome, I. (1988). A complication of intercostal insertion of chest drain (letter). *Br. J. Anaesth.*, **61**, 642–643.
[13] McConaghy, P. M. and Kennedy, N. (1995). Tension pneumothorax due to intrapulmonary placement of intercostal chest drain. *Anaesth. Intensive Care*, **23**, 496–498.

### COMMENTARY

Merely performing a right hepatic lobectomy without diaphragmatic involvement can result in a right pleural effusion which may take several days to make its presence known. This was a regular occurrence in the days of thoraco-abdominal incisions and therefore some authorities routinely used chest tubes with these incisions. Pleural effusions have become relatively infrequent with bilateral subcostal incisions and treatment in these cases by thoracentesis is usually successful.

Pain and respiratory compromise are known difficulties of chest tubes. Blocking and kinking of the tubes are rare, if serious complications, and any successful effort to avoid these problems should be welcomed.

However, in my experience, diaphragmatic involvement by liver tumors is of serious prognostic significance. Because symptoms can be severe, especially intractable pain, I do not fault the authors for performing an *en bloc* dissection, but as there is no follow up, I cannot but wonder about the long term outcome. Nonetheless, pain can be reduced by diaphragmatic resection, and significant palliation can be achieved. But a limited lifespan should not be marred by continuing pain from a chest tube, and Chung *et al.*, have shown a way of qualitatively improving the postoperative period in avoiding this step by intraoperatively clearing supradiaphragmatic fluid.

Prof. W. John B. Hodgson, M. D.
Prof. W. J. B. Hodgson
Department of Surgery
The Brooklyn Hospital Centre
121 DeKalb Avenue
Brooklyn
New York 11201
USA

